# ‘*Co‐Production Is Caring*’: Young People's Reflections on Responsible and Dialogic Co‐Production in Youth Mental Health

**DOI:** 10.1111/hex.70488

**Published:** 2025-11-25

**Authors:** Josimar Antônio de Alcântara Mendes, Ayan Mahamud, Sarah Doherty, Mathijs Lucassen, Joanna Lockwood, Ellen Townsend, Chris Hollis, Marina Jirotka

**Affiliations:** ^1^ Responsible Technology Institute, Department of Computer Science University of Oxford Oxford UK; ^2^ Sprouting Minds (Young People Advisory Group—Digital Youth Programme), Queen's Medical Centre University of Nottingham Nottingham UK; ^3^ Northampton Square City St George's, University of London London UK; ^4^ Queen's Medical Centre University of Nottingham Nottingham UK

**Keywords:** co‐production, dialogic, Responsible Research and Innovation, youth mental health

## Abstract

**Background:**

While co‐production is increasingly emphasised in youth mental health research, few studies have explored how young people themselves conceptualise and evaluate *responsible and dialogic* co‐production. Understanding young people's perspectives is essential to ensure that participation is meaningful and protective, rather than tokenistic or exploitative. This paper offers a retrospective reflection on a 3‐year UK youth mental health programme that embedded youth involvement and co‐production from the outset, at multiple levels (research participation, advisory and leadership).

**Objective:**

This study examines how young people involved in a UK youth mental health research articulate, from their own perspective, what counts as ‘meaningful co‐production’, centring its responsible, relational and dialogic dimensions.

**Design:**

A Qualitative Secondary Analysis was undertaken, applying Reflexive Thematic Analysis to explore patterns and meanings in participant accounts.

**Setting and Participants:**

Data comprised responses from five young people (three females and two males; *M* = 21 years, SD = 2.74) via an online open‐ended survey, and a focus group with eight young people (seven females and one male; *M* = 25.63 years, SD = 3.03). All participants had lived experience and were under 24 years old when they began their involvement in the youth mental health research programme on which this study is based.

**Results:**

Two central themes emerged: (1) ‘We just want to be cared about’: Co‐production is caring and (2) ‘Please, show up as a person, not as a “researcher”’: Co‐production as a dialogic process. Young people emphasised that meaningful co‐production, in youth mental health, is relational and affective (i.e., rooted in emotional care, mutual respect, flexibility and dialogue) and that the living experience of mental health is continuous and demands sensitivity.

**Discussion:**

These insights challenge procedural or ritualistic approaches to participation. Instead, they foreground care, reflexivity, power sharing and researcher presence as ethical prerequisites of co‐production. The results align with Responsible Research and Innovation (RRI) principles, highlighting the need to embed structural supports for emotional safety and relational engagement from the outset.

**Conclusions:**

Meaningful co‐production in youth mental health research requires embedding relational ethics into design and practice, ensuring young people are engaged as whole persons and partners. This model moves beyond procedural inclusion towards genuinely participatory research.

**Patient or Public Contribution:**

Young people with lived experience co‐designed the study materials, co‐facilitated the focus group, contributed to the interpretation and co‐authored the manuscript—ensuring that their perspectives are central to the study. Young people with lived experience of mental health were central to this study. Members of the programme's Young People's Advisory Group (YPAG) contributed to the design of the survey and focus group materials, ensuring they were youth‐friendly and relevant. Two YPAG members co‐facilitated the focus group alongside the first author, actively shaping the data collection process. Their insights and perspectives also informed the interpretation of the data, particularly in refining how themes captured relational and dialogic aspects of co‐production. Additionally, these two YPAG members are co‐authors of the manuscript, contributing to the conceptual framing, reflexivity and clarity of the results, ensuring that the work authentically reflects youth perspectives.

## 1 Introduction

Over the past decade, co‐production has gained prominence in health and social care research, especially within the youth mental health field [1, 2, 3, 4, 5, 6, 7]. Co‐production can be understood as a collaborative process in which professionals, service users and communities work together to shape knowledge and practice by balancing power and sharing decision‐making throughout the process [8, 9, 10, 11, 12, 13, 14, 15].

In health research, co‐production is recognised for enhancing relevance, legitimacy and impact by valuing lived experience, sharing power and building meaningful working relationships [5, 8, 12, 15, 16, 17, 18]. Within the youth mental health field, the literature commonly associates co‐production with terms such as ‘co‐design’, ‘co‐research’ and ‘consultation’ [19]—in a rapid review, O'Mara‐Eves et al. [11] likewise noted that ‘co‐design’ and ‘co‐research’ are frequently invoked in publications addressing co‐production processes. However, amid this ‘participatory zeitgeist’ [12, p. 247], the proliferation of ‘co‐’ labels can obscure critical distinctions (e.g., who is involved, how and to what end), risking conceptual conflation, or ‘cobiquity’ [20, p. 2], and sidelining considerations of power dynamics and epistemic justice [11, 18, 20, 21, 22].

Against this backdrop of expanding practice, but persistent conceptual and power‐related ambiguities, young people are increasingly expected to contribute across all stages of mental health research, from conception and design to eventual dissemination [3, 4, 5, 7, 13, 18, 22, 23, 24, 25, 26, 27, 28, 29, 30]. However, meaningful youth engagement can be challenged by barriers such as fragile trust and the absence of welcoming, dialogic spaces [11, 18, 26]. Conversely, facilitators include safe, informal spaces, accessible language, and an element of enjoyment to foster inclusion and reduce power imbalances [13, 16, 26, 28, 29, 31].

In addition, researchers should weigh the benefits of participation against potential distress (particularly when lived experience is involved) and remain alert to how approaches may drift along a continuum from ‘consumerist’ (instrumental, top‐down) to ‘democratic’ (rights‐based, inclusion‐ and autonomy‐oriented) over the course of collaboration [3, 7, 22, 32]. These caring‐related issues intersect with other important safeguards in youth mental health research projects: young people's well‐being can fluctuate over time, necessitating flexible modes and tempos of involvement [7, 8, 26, 33]. Without careful attention to these issues, the rhetoric of co‐production can mask persistent inequalities [20] and expose young people to risk, potentially causing unintended harms [8, 27, 33]—an illustrative example of this is the Aotearoa New Zealand experience where Indigenous Māori researchers have reinforced the importance of the ‘caring dimension’ in research with *rangatahi* (youth) which is enacted through the notion of ‘*Manaakitanga*’ (i.e., nurturing, caring, generosity and recognising others' dignity in interpersonal interactions) [34, 35].

While meaningful involvement benefits researchers and participants [12, 26, 36], exploitative and tokenistic practices may persist [37, 38, 39, 40]. These arise when young people's contributions are undervalued, dialogue is constrained, and inclusive spaces are lacking [11, 18, 36]. Without supportive relationships and clear expectations, the collaboration with young people can risk reinforcing hierarchies [4, 36, 41]. Hence, co‐production in youth mental health should be acknowledged as a dialogic, relational process grounded in communication, mutual learning and shared understanding [13, 33, 36, 42, 43]. This approach foregrounds relationship building, requiring planning, flexibility, reflexivity and power sharing built on trust, role clarity and reflective space [13, 26, 44, 45, 46]—yet these ethical and interpersonal dimensions are often overlooked.

In this scenario, two gaps persist regarding participation in youth mental health. Firstly, reporting of young people's involvement remains inconsistent, limiting cumulative learning about benefits, impacts and practical challenges [21, 24, 27]. Secondly, comparatively few accounts centre young people's own reflections on what makes co‐production meaningful and non‐tokenistic for them; most literature privileges implementation case notes or researchers' perspectives [37, 38, 39]. Given the evidence that youth involvement can improve research processes and outcomes [4, 11, 18] and that authentic engagement hinges on relational empowerment, fluidity and flexibility [13, 46], there is a clear need to amplify youth‐led conceptualisations (especially those framing co‐production as a responsible, relational process from young people's perspective) and to strengthen evaluation of participatory practices accordingly.

In light of these issues, the reflections presented in this paper are drawn from Digital Youth (https://digitalyouth.ac.uk/), a 3‐year research programme that embedded youth involvement at multiple levels [11, 47]: *transformative* (young people sharing decision‐making and reciprocal expertise that reoriented research purposes—e.g., we had a Young People's Advisory Group (YPAG) and Youth Co‐Chairs^1^ shaping programme priorities and decisions from the outset); *intermediate* (young people involved in active participation and mutual recognition with limited decision‐sharing—e.g., young people helped us to co‐develop and champion research materials); and *descriptive* (young people informing/engaging without sharing decisions—e.g., we had consultation/feedback from young people at specific stages and across different work packages). We understand this layered design responds to calls to open institutional decision‐making and include plural voices in shaping research agendas and practices [11, 18, 48, 49].

In the Digital Youth, we understood co‐production as contiguous with, yet distinct from, Patient and Public Involvement (PPI), and we positioned Responsible Research and Innovation (RRI) as a complementary governance frame to ensure both meaningful and protective involvement. In this configuration, PPI integrated young people patient/public voices in research; co‐production advanced this by redistributing decision‐making and cultivating relational practices in day‐to‐day work with young people; and RRI extended both by foregrounding anticipation (e.g., scrutinising potential harms/benefits and unintended effects), inclusion (e.g., broad, meaningful stakeholder participation), reflexivity (e.g., on purposes, values and positionalities), and responsiveness (e.g., adapting trajectories in light of stakeholder concerns) [8, 27, 33, 50, 51]. In the end, both PPI and RRI served to amplify young people's voices and promote ethically sound, democratic and protective involvement [27, 52, 53, 54], which is pivotal to upholding the core values of co‐production, particularly within the youth mental health field [3, 11, 18, 22, 47].

In this context, this study examines how young people involved in the Digital Youth programme reflect retrospectively on responsible and dialogic co‐production, using Qualitative Secondary Analysis (QSA) of survey and focus group data. The research questions were: (1) how do young people retrospectively describe the values and practices that constitute meaningful co‐production in youth mental health? and (2) which relational, caring and dialogic factors support or hinder non‐tokenistic engagement in youth mental health?

## 2 Materials and Methods

This study adopted a QSA approach, which offers a robust framework for reinterpreting existing data to address new questions or deepen insight [55, 56, 57]. We selected QSA to enable a critical, reflexive exploration of young people's experiences of co‐production based on their real‐world involvement in a youth mental health research programme. Specifically, we conducted a retrospective, reflexive analysis of young people's accounts of involvement and co‐production, focusing on how participation was enacted and experienced. Rather than a summative evaluation of the programme's effectiveness, this study offers a retrospective appraisal of involvement and co‐production from the perspectives of the young people involved.

The two qualitative datasets used for this QSA served complementary purposes. The first comprised an anonymous online survey with detailed open‐ended items, exploring how RRI principles were understood and enacted across the programme, and it gathered perspectives from both researchers and young people across work packages. The second comprised a focus group with YPAG members, convened to reflect on the survey results and on a young‐people‐friendly version of those results championed by YPAG members—the second and third authors of this article. The breadth of the survey responses, coupled with the depth and dialogic nuance of the focus group, provided rich, complementary material (offering both cross‐programme coverage and situated, collective reflection), which together justified and strengthened the rationale for conducting this QSA—see Supporting Material 1 for the survey and focus group questions.

### 2.1 Participants

Five young people (three females and two males; *M* = 21 years, SD = 2.74) involved at *intermediate* (e.g., co‐developing and championing study materials) and *descriptive* levels (e.g., providing consultation/feedback at specific stages) within the programme completed the survey. Eight YPAG members took part in the focus group (seven females and one male; *M* = 25.63 years, SD = 3.03); all were involved in a *transformative* level (shaping programme priorities and decisions from the outset). As this is a retrospective study, all YPAG members were 24 years old or younger when they began their involvement as ‘young people’ (as defined by [58]) in the programme.

All young people had prior involvement in mental health projects and identified as having lived experience. Because YPAG members also participated at intermediate and descriptive levels within the programme, some survey respondents likely also took part in the focus group. This overlap is a methodological strength, allowing consolidation and elaboration of perspectives across complementary sources [59, 60].

We acknowledge that treating youth co‐researchers, including YPAG members, as ‘participants’ is contested, given their constitutive roles in design, deliberation and decision‐making [11]. However, as also noted in the literature, organisations and journals often lack clear mechanisms to recognise youth co‐research contributions (particularly when underage individuals are involved), so ethical review and formal participant procedures are frequently required to protect young people and enable transparent reporting [11, 18]. Accordingly, for this retrospective study, we sought ethics approval and applied participant safeguards (consent, confidentiality and optional non‐disclosure of identifiers) to YPAG contributors whose reflections are reported here, while continuing to recognise their substantive co‐research roles elsewhere in the programme.

### 2.2 Materials, Procedures and Data Collection

Data were collected via an online open‐ended survey and a semi‐structured focus group. The survey, examining young people's co‐production experiences and views on RRI, was distributed digitally to programme participants and completed asynchronously (see [33]). A youth‐friendly video summarising this study's results (click here to watch) was then produced by two YPAG members, A.M. and S.D. (the second and third authors). As young people, A.M. and S.D. had full autonomy over the script, creation of the clip and voice‐over; researcher support was available but not required. The first author's sole role was to review the final cut to ensure alignment with the survey results; no changes were made.

An online focus group with the other YPAG members followed, which was designed to critique and reflect on the survey findings as well as the video. A.M. and S.D. were free to decide how to conduct the session, and they led an open, dialogic discussion; the first author provided light logistical support only and asked a couple of questions at the end. After reviewing the session notes and recording, the first author approached A.M. and S.D. to propose the scope of this current article, seek their views on its relevance and invite their participation as co‐authors. The 75‐min session generated rich material well‐suited for Reflexive Thematic Analysis (RTA).

### 2.3 Data Analysis

Focus group data can be analysed individually, as a group, or by examining participant interactions [61, 62]. Given our focus on personal experiences of co‐production, we chose the individualised approach. While this does not capture group dynamics, it allowed us to explore the diversity of perspectives on youth mental health. Hence, in this study, each participant, whether from the survey or the focus group, was treated as the ‘unit of analysis’ [28].

Our analysis followed Braun et al. [63] and Braun and Clarke's [64] guidelines, whose RTA provides a flexible yet robust method for interpreting meaning patterns. RTA highlights the researcher's active role, shaped by their positionality and engagement with the data. The first author conducted the analysis and engaged in reflexivity^2^ regarding his own background, motivations and experience in youth mental health and participation [64, 67, 68]. All authors contributed to the analyses (e.g., reviewing and defining themes). Although the project was conducted in the United Kingdom, the team drew on international perspectives when interpreting the results (e.g., the first author's child rights and youth co‐production experience in Brazil and M.L.'s work with community‐based organisations for youth in Aotearoa, New Zealand). The first author's reflexivity account is available in Supporting Material 2. A.M. and S.D. were involved in the interpretation and discussion of the RTA's results.

RTA is a flexible, non‐prescriptive method shaped by the dataset and analytic goals [64, 67, 69, 70], with no fixed rule on the number of themes; instead, analytic depth, coherence and relevance are prioritised [63, 64, 71]. Our RTA followed five phases [28, 72]: (1) *familiarisation*, in which the first author engaged with the survey responses and full focus group transcript, making preliminary notes and identifying patterns; (2) *open coding*, a line‐by‐line analysis developing inductive semantic and latent codes across the dataset—18 initial codes generated, see Supporting Material 3; (3) *generating initial themes*, clustering codes into broader candidate themes and features (i.e., sub‐themes) through recursive and interpretive engagement with the data—2 Candidate themes and 6 features, Supporting Material 4; (4) *reviewing and defining themes*, refining, naming and relating them to the research questions; and (5) *anchoring themes*, mapping connections between themes and participant contributions.

## 3 Results

Young people are identified as ‘YP‐FG’ (Focus Group) or ‘YP’ (Survey), with numerical identifiers (e.g., YP1‐FG and YP9‐S). Table 1 outlines the themes and features (i.e., sub‐themes), each anchored in young people's accounts. This anchoring enhances transparency and trustworthiness by tracing interpretations to the data and showing the relational depth of contributions [28, 72].

**Table 1:** Themes, features and their anchoring on the data.

Theme	Anchoring
**Theme 1: ‘** ***We just want to be cared about***’**: Co‐production is caring**	
**Feature 1.1**—‘*This is not just data; this is real people's lives*’: being sensible about ‘lived experience’	YP1‐FG, YP2‐FG, YP4‐FG, YP5‐FG
**Feature 1.2**—‘*We still are going through those experiences*’: recognising ‘lived experience’ as a common and ongoing issue	YP1‐FG, YP2‐FG
**Feature 1.3**—*Making young people feel heard and important to the research*: co‐production as a responsive, safe and inclusive space	YP1‐FG, YP2‐FG, YP3‐FG, YP4‐FG, YP5‐FG, YP10‐S, YP13‐S
**Theme 2: ‘** ***Please, show up as a person, not as a “researcher*** **”’: Co‐production as a Dialogic Process**	
**Feature 2.1**—‘*I think we've learned a lot from each other*’: room for exchange and learning	YP1‐FG, YP2‐FG, YP3‐FG, YP4‐FG, YP5‐FG, YP9‐S, YP12‐S
**Feature 2.2**—‘*When does age not really matter and it is more about the ideas?*’: challenging the ‘Us vs. Them’ culture in co‐production	YP1‐FG, YP2‐FG, YP5‐FG, YP7‐FG
**Feature 2.3**—‘*Breaking down some barriers*’: aiding the dialogic process	YP1‐FG, YP2‐FG, YP3‐FG, YP4‐FG, YP5‐FG, YP6‐FG

This RTA captured young people's nuanced, context‐specific perspectives on co‐production, highlighting the interdependence between its ‘caring’ and ‘dialogic’ dimensions. The two overarching themes reflect the focus on exploring young people's lived experiences in depth.

### 3.1 Theme 1: ‘*We Just Want to be Cared About*’: Co‐Production Is Caring

This theme highlights young people's view of co‐production as relational and affective, rooted in care, respect and sensitivity. They rejected transactional involvement, instead stressing the need for emotionally responsive engagement that acknowledges lived experiences and vulnerabilities. For them, care, in this context, is not peripheral but central—embodied in empathy, attentiveness, flexibility and genuine concern for young people's well‐being.

Feature ‘*This is not just data; this is real people's lives*’: being sensible about ‘lived experience’ (1.1) reports that young people repeatedly emphasised the importance of treating their participation not as data extraction, but as a human interaction grounded in recognition of their lived experiences. ‘*This* [mental health] *is not just, obviously, data, this is like real people's lives*’, noted YP1‐FG, highlighting how reductive approaches risk dehumanising those involved. The same participant added: ‘*we're all sensitive human beings, you know, we just want to be cared about*’—underscoring that respectful involvement must extend beyond methodological rigour to encompass emotional sensibility. Some acknowledged that caring was evident from the start: ‘*I had a meeting with the PPI coordinator and it was just asking like questions that no one else really would ask* [e.g., how can we support your involvement in this project?—considering their “lived experience” with mental health] *(…) I went with no expectations. But yeah, I definitely felt like that cared, being cared for*’ (YP4‐FG).

This sensibility is especially vital given the nature of the topics addressed in youth mental health projects. As YP2‐FG remarked, ‘*it* [mental health] *is a very real subject for everyone involved (…) I think it's easy to forget that you're dealing with humans*’. There was a shared concern about whether empathy could coexist with research objectives, expressed in the question: ‘*you're also dealing with sensitive topics and it's like, how do we take a more empathetic approach (…) but still achieving those objectives?*’ (YP2‐FG). YP4‐FG noted that their previous research experiences had lacked such sensitivity, describing it as ‘*a nice change*’ that other members of the project asked these kinds of questions. The emotional toll of participation was also acknowledged: ‘*I think especially with young people with mental health, it can be hard to find the time and if you have not only the time, but if you have mental health challenges that can get in the way, you know (…) there was some real significant reasons why I couldn't* [continue to] *be involved* [in the co‐production]’ (YP5‐FG).

Feature ‘*We still are going through those experiences*’: recognising ‘lived experience’ as a common and ongoing issue (1.2) showcases that lived experience is not static or bygone—it remains active and unfolding: ‘*we often talk about lived experience like it's something in the past and we kind of forget that sometimes … well, most of the time, we are still living it (…), going through those experiences*’ (YP2‐FG). For this reason, they felt that co‐production must account for this continuity and avoid assumptions of a supposed resolution or that some level of detachment is viable. As YP2‐FG pointed out, ‘*like the term “lived experience” … everyone has lived experience of mental health; like we all have mental health* [challenges] *and everyone will and has or will* [be] *finding things tough in one area of their life*’.

The tension between structured processes and emotional realities was also noted. ‘*Sometimes it's very easy to forget* [that] *we are dealing with lived experience* [people]’, said YP2‐FG, referring to how procedural constraints may inadvertently overlook young people's ongoing struggles. YP1‐FG echoed this, affirming that ‘*it's important (…) still taking care of those* [lived experiences] *… because they* [young people] *are still living it*’.

Feature *Making young people feel heard and important to the research*: co‐production as a responsive, safe and inclusive space (1.3) makes a case that, for co‐production in youth mental health to be meaningful, it must be inclusive, flexible and compassionate, respecting individual needs. As YP10‐S succinctly put it, the goal should be ‘*making the young people feel heard and important to the research*’. This was linked not only to emotional well‐being but also to sustained involvement. YP5‐FG stated: ‘*I didn't have the luxury of that* [mental health challenges] *not being part of my life…*’, consequently, they perceived the co‐production space as somewhere they ‘*could come back to*’ when mental health challenges made consistent involvement difficult, offering ‘*confidence from being around other people*’.

Young people appreciated formats that allowed for various types of involvement, such as engaging in chatting, speaking after sessions, or receiving information in ways tailored to their preferences: ‘*leaving the right amount of space for everyone to have that chance* [to speak out]’ (YP4‐FG) and ‘*that kind of patience with everyone … researchers, lived experience* [people], *whoever it is … it needs to be incorporated* [in youth mental health research]’ (YP1‐FG). Communication was central to this sense of care. YP1‐FG reflected on how a lack of feedback could diminish their motivation: ‘*I think when you're kind of not communicated back*, [you] *kind of just like: “OK, what's the point?” kind of thing. Whereas, obviously, if you know that as much as you care about the project people care about you too, you want to give more to it*’.

Acknowledgement within the co‐production process was contrasted with past experiences. YP13‐S remarked: ‘*I have felt really valued* [in this project] *compared to my experience in the NHS or other services which are conducting PPI activities*’. Others remembered that ‘*everyone* [in the project] *had like well‐being plans …* [and] *conversations of like how you prefer to be communicated with*’ (YP2‐FG). These acts of inclusion were not seen as minor accommodations but as central to co‐production work where team members genuinely care for each other.

### 3.2 Theme 2: ‘Please, Show Up as a Person, Not as a “Researcher”’: Co‐Production as a Dialogic Process

This theme reflects young people's view of co‐production in youth mental health as a relational and reciprocal dialogue built on shared understanding. Young people valued interactions grounded in mutual respect, informality and authentic engagement. For them, co‐production is most effective when researchers ‘*show up as a person*’ (i.e., when they have a ‘humanistic presence’), bringing presence, humility and a genuine commitment to collective meaning‐making.

Feature ‘*I think we've learned a lot from each other*’: room for exchange and learning (2.1) captures how young people valued the dialogic and interpersonal aspects of co‐production. Informal exchanges (such as humour, small talk or check‐ins) played a vital role in building trust and dismantling hierarchical barriers: ‘[having] *the small talk, the chit chat and just like, show up as a person, not as a “researcher” (…) I think that for me has helped*’ (YP2‐FG). This informality allowed young people to feel more comfortable and encouraged open sharing. YP3‐FG reflected that ‘*having the human element makes so much difference, especially through in‐person meetings or events, as well as through small talk and jokes. It makes you see researchers as just like other people who want to work with you*’. This interpersonal approach can enable freer thinking: ‘*it makes the conversation much more natural and so you're able to think about things more freely and effectively*’ (YP3‐FG).

Mutuality and shared investment were also underscored: ‘*there's more to it* [the co‐production process] *and I think we've learned a lot from each other*’ (YP1‐FG). Others emphasised the emotional dimension of this process: ‘*just that constant kind of like relationship building … it's more than just “I need something from you and you need something from me*”’ (YP1‐FG). YP12‐S added: ‘*my collaboration within this programme has been hugely beneficial for myself, and hopefully my contributions have had a positive impact*’. The space for respectful disagreement was equally valued: ‘*I think having that opportunity to disagree is something that you don't often get* [in health‐related projects]’ (YP3‐FG).

Feature ‘*When does age not really matter and it is more about the ideas?*’: challenging the ‘Us vs. Them’ culture in co‐production (2.2) focuses on how young people resisted the dichotomic distinctions often constructed between ‘young people’ and ‘researchers’. For them, these oppositions risked undermining the essence of co‐production in youth mental health. As YP2‐FG remarked: ‘*we're young people and you're a researcher (…) and actually, when does age not really matter and it is more about the ideas?*’. They noted that the boundary‐making discourse (i.e., ‘*we* [are youths and] *have lived experience and you don't*’) oversimplified relationships and missed the nuance of collaboration—when reviewing these results, one of the researchers and authors of this paper reflected: ‘*some of us have had lived experience as a young person with mental health challenges. It makes me wonder whether I* [as a researcher] *should have shared more of that during huddles* [for instance, so that these divisions could be eased]’. In this context, young people advocated for relational bridges that helped dismantle artificial divisions. The idea of YPAG Co‐chairs was cited as one such bridge: ‘*I think the whole idea of having like the Co‐chairs was to have that bridge and that gap between the “them and us.” (…) it's just like being able to speak both languages*’ (YP2‐FG); ‘*I think having a Co‐chair role is really important … someone you can contact who's our age but also has those links has felt really valuable*’ (YP5‐FG).

YP1‐FG highlighted how conversations that transcended the dichotomic roles were ‘*really enlightening and really, really good for us young people (…) but also researchers (…) to hear it from different perspectives*’. At the same time, the practical need for some of these categories was acknowledged: ‘*those distinctions are also what enable researchers to try and incorporate young people more. So if you didn't have that distinction, then how would you start* [the work]?’ (YP7‐FG).

Feature ‘*Breaking down some of those barriers’:* aiding the dialogic process (2.3) brings attention to the intentional structures and formats that foster openness and accessibility. Young people spoke positively of mechanisms like regular huddles (i.e., periodical whole programme meetings with most of the team members present) and targeted away days as spaces that built rapport and helped them connect with researchers on a personal level: ‘*The huddles have been really good with breaking down some of those barriers in communication, like building those relationships, being able to see people in real life and get a feel for, like their personality*’ (YP2‐FG). This physical and relational proximity helped demystify roles and promote engagement: ‘*you can kind of get a sense of, like, what they* [researchers] *are like … I feel like that's helped a lot of people be more engaged in the research*’ (YP2‐FG).

Opportunities for asynchronous contributions were equally valued, such as surveys and reviewing materials for feedback (YP4‐FG). Informal interactions, such as away days, seemed to facilitate meaningful engagement as well (YP1‐FG). Young people also valued in‐person meetings as they ‘*make it so much easier to engage with each other and build strong relationships, can feel the good vibes easier*’ (YP6‐FG).

## 4 Discussion

The results highlight that co‐production in youth mental health should not be merely procedural but deeply relational and grounded in attentiveness, mutual respect and emotional care. When young people are actively engaged in collaborative processes, they ‘*just want to be cared about*’, urging researchers to treat mental health lived experience as part of ‘*real people's lives*’. This reflects RRI's responsiveness principle, which calls for research attuned to young people's lived experiences, especially in emotionally complex contexts like youth mental health [8, 33, 51, 73]. Complementary to this, it also resonates with Indigenous wisdom as reinforced by Māori researchers (e.g., [35]), such as the Māori concept of *Manaakitanga*, which emphasises nurturing, care, generosity and the upholding of others' dignity through relational practice. In the context of co‐production, concepts that underscore the ethical responsibility of researchers to nurture respectful, inclusive relationships that value young people, not merely for their contributions, but as whole persons whose well‐being must be safeguarded [34, 35].

Within this care‐centred framing (grounded in young people's call to be ‘*cared about*’), the results indicate that responsible co‐production in youth mental health could benefit when the ‘caring dimension’ is acknowledged from the outset—not as an added feature, but as a guiding principle throughout planning, implementation and follow‐up. This entails prioritising well‐being and responding sensitively to the ongoing realities of young people with lived mental health experience [8, 36, 42]. Hence, co‐production in youth mental health should not be reduced to impersonal and acritical consultation or superficial representation; it must be a safe, inclusive and emotionally responsive process, with built‐in flexibility and support from the start.

In line with RRI's anticipation principle, potential harms and unintended consequences must be carefully considered in advance and operationalised through co‐production practices that foreground care, inclusion and power sharing [27, 33, 50]—which also aligns with the current guidelines for co‐production (Hawke et al. 2022 [4, 11, 18, 44, 47]). As such, safeguarding, check‐in systems and accommodations for fluctuating mental health should not be add‐ons but integral to responsible youth mental health co‐production practices [8, 36, 41, 42]. In our programme, this can be exemplified by the work package where researchers and young people co‐designed a self‐harm support card‐sorting app: researchers anticipated potential risks and implemented wellness plans, pre‐session risk check‐ins, trigger warnings and structured debrief/follow‐up processes (for more details, see [8]).

The results reinforce that, in youth mental health co‐production, ‘care’ emerges through relationship building. Young people linked feeling ‘*cared for*’ not just to actions like being heard or checked in on, but to researchers ‘*showing up as a person*’ (i.e., adopting a humanistic, authentic and relational presence). This aligns with literature emphasising rapport built through approachable communication, shared motivations and emotional attentiveness in youth co‐production settings [13, 14, 41, 42, 44]. As Smith et al. [14] note, relational ethics are lived through evolving, situated practices, not abstract commitments. However, we understand that care in youth mental health co‐production goes beyond interpersonal warmth; it requires critical reflection on how young people's lived experience is engaged with. Young people rejected being treated as mere data sources, instead emphasising that lived experience is ongoing, emotional and dynamic. Reducing it to instrumental knowledge dehumanises young people and undermines the ethical core of co‐production, especially in youth mental health [8, 14, 41, 46]. Thus, responsible co‐production, in this context, involves acknowledging young people's vulnerabilities and building in emotional support mechanisms [73]—a requirement consistent with RRI principles of reflexivity and inclusion [8, 27, 33].

Central to these relational processes is the dialogic nature of co‐production in youth mental health. Our results indicate that young people perceived co‐production as meaningful when it was grounded in informal, mutual learning spaces that encouraged collaboration and reflection. Dialogic interaction, including space for disagreement, helped dismantle hierarchical roles and foster a sense of shared ownership. This reflects Wegerif's [74] view of dialogic spaces as opening, broadening and deepening through the inclusion and negotiation of diverse perspectives. Sustained by trust and intersubjectivity, such spaces enable young people to fully engage, fostering creative thinking and psychological safety [13, 41, 75, 76]. Yet, dialogic care also requires researchers to critically reflect on their assumptions and beliefs about youth and their capacity for agency. Without this reflexivity, participation risks becoming tokenistic and reinforcing pre‐existing power dynamics [4, 5, 11, 13, 18, 22, 29, 42, 47, 77]. It is known that co‐production may reproduce hierarchies and exclusions if not grounded in meaningful, equitable collaboration [4, 5, 9, 11, 13, 18, 22, 40, 41, 78]. We understand that RRI principles encourage such reflexive interrogation by explicitly asking researchers to consider their positionality, the context of involvement, and the broader societal and ethical implications of their work [8, 27, 33, 51].

Our results also reveal the challenges of the perceived binary between ‘young people’ and ‘researchers’. Young people valued relational bridges, like the Co‐chair role, that fostered equitable communication and shared decision‐making. Dismantling such binaries, however, demands confronting power imbalances embedded in institutional, cultural and interpersonal contexts. Power is not only hierarchical but also intersectional, shaped by transversal factors like age, class and health status [42, 73]. Accordingly, fostering reciprocal and inclusive relationships requires acknowledging these dynamics, alongside sustained time, effort and intentional design [5, 13, 20, 41, 44].

Overall, both themes also foreground the emotional, personal and academic labour involved in youth mental health co‐production. For young people with lived experience, participation requires vulnerability, time and investment. For researchers, it demands reflexivity, flexibility and sustained relationship work. These realities must be acknowledged by institutions and funders, who too often assume that co‐production can be seamlessly integrated into existing timelines and budgetary frameworks [79]. As Watson et al. [36] argue, responsible co‐production requires dedicated funding, skilled facilitation and flexibility to meet diverse needs. Without institutional support, the burden risks falling unfairly on young people or individual researchers.

### 4.1 Strengths and Limitations

This study foregrounds care as a core feature of co‐production in youth mental health and links it explicitly to RRI by showing how anticipation, inclusion, reflexivity and responsiveness can be operationalised to support non‐tokenistic, protective participation. Drawing on a multi‐year programme, it specifies practical mechanisms (e.g., well‐being planning, proactive check‐ins, trigger warnings, structured debriefs and flexible participation options), moving beyond general calls to avoid tokenism (e.g., ‘include the youth voice’, ‘create safe spaces’, ‘build trust’, ‘share power’ and ‘be inclusive and responsive’) towards actionable guidance. Youth partnership was embedded throughout (e.g., co‐design of materials, co‐facilitation of data generation, involvement in interpretation and co‐authorship).

The work is situated in a single UK programme with a modest sample for this study, which may limit transferability. Although the wider YPAG group was diverse, most focus‐group contributors were non‐Black females, and additional intersectional characteristics were not systematically collected. As a retrospective QSA, accounts may be influenced by recall or social desirability, and the study does not evaluate programme outcomes or compare modalities across work packages.

## 5 Conclusion

This study underscores that young people conceive co‐production in youth mental health research as a relational, affective and dialogic process grounded in care, empathy and mutual respect. Responsible co‐production, they argue, extends beyond mere inclusion and requires emotional responsiveness, structural flexibility and sustained attentiveness to lived experience. In this sense, care should not be peripheral as it shapes how relationships are built, how power is shared and how young people feel safe and valued in research.

Young people stressed that lived experience is ongoing and cannot be reduced to extractable data. Hence, meaningful and responsible involvement in youth mental health entails acknowledging vulnerability, providing support and engaging with young people as whole persons. This reinforces the call for a shift from instrumental approaches to co‐production grounded in relational ethics and genuine dialogue, especially in the field of youth mental health. For young people, dialogic co‐production thrives when researchers present themselves as ‘people’—that is, ready to listen, reflect, disagree and co‐learn in spaces built on trust and informality.

Practically, aligning co‐production in this field with RRI principles means anticipating potential harms, ensuring inclusion, practising reflexivity and remaining responsive to concerns throughout the project. Measures such as well‐being planning, proactive check‐ins, trigger warnings, structured debriefs and flexible participation options exemplify how care can be operationalised to protect and empower youth partners.

As already signposted by other authors, organisations and funders must acknowledge the emotional, intellectual and relational labour co‐production demands, especially in youth mental health, by resourcing skilled facilitation and training, allowing flexible timelines, and enabling genuine power‐sharing. Without such structural support, care and adaptation may fall unfairly on young people or individual researchers. When care is embedded systemically, as well as interpersonally, co‐production can become a more responsive, inclusive and reflexive practice that creates dialogic spaces in which young people are heard, valued and respected as true partners in knowledge production.

## Author Contributions

**Josimar Antônio de Alcântara Mendes:** conception, study design, collection, data analysis, writing and editing. **Marina Jirotka:** conception, study design, writing and editing. **Ayan Mahamud:** study design, collection, writing and editing. **Sarah Doherty:** study design, collection, writing and editing. **Mathijs Lucassen:** study design, data analysis, writing and editing. **Joanna Lockwood:** writing and editing. **Ellen Townsend:** writing and editing. **Chris Hollis:** writing and editing.

## Ethics Statement

Both the survey and focus group received ethical approval from the University of Oxford's Computer Science Departmental Research Ethics Committee (Ref: CS_C1A_23_033)—granted on 14 January 2024.

## Consent

Participation was voluntary and based on informed consent, with safeguards ensuring anonymity. As all participants were over 18 years old, parental consent was not required. Given the sensitive nature of the discussions and participants' preferences, full transcripts were not shared; however, anonymised excerpts and supporting materials were provided to ensure transparency.

## Conflicts of Interest

The authors declare no conflicts of interest.

## Supporting information


**Supporting Material 1** – Survey and Focus Group Questions.

**Supporting Material 2** – Reflexivity.

**Supporting Material 3** – Initial Codes.

**Supporting Material 4** – Candidate Themes.

## Data Availability

Given the sensitive nature of discussions and participant preferences, full transcripts are not available, but anonymised excerpts and Supporting Materials ensure transparency.

## Endnotes

1 Within the Digital Youth Programme, ‘Co‐chair’ was a formal role for young people who shared leadership with adult researchers. They help set agendas, facilitate meetings, represent youth perspectives and ensure that discussions and decisions reflect intergenerational equity. By doing so, Co‐chairs move beyond tokenism, redistribute power and position young people as genuine partners and leaders in youth mental health and digital innovation.

2 Reflexivity is an ongoing process of the researcher's self‐reflection that surfaces awareness of actions, feelings and perceptions throughout the analysis [65, 66]. It seeks to recognise how the phenomenon under study affects the researcher, what this reveals about them and how it intersects with their history, assumptions and values. Its purpose is to examine how the researcher's subjectivity may shape the analysis by identifying potential perspectives that enter the investigation and interpretation of the phenomenon. Rather than assuming sterile neutrality or a strict orthogonality between observer and object, reflexivity acknowledges non‐neutrality and traces its implications for investigating and understanding the object of enquiry.
