# Supplementary material for: ‘Co‐Production Is Caring’: Young People's Reflections on Responsible and Dialogic Co‐Production in Youth Mental Health
Source: Health Expect. 2025 Nov 25;28(6):e70488. doi: 10.1111/hex.70488 (PMC12646113; doi:10.1111/hex.70488)
Supplement: Supplementary file 1 — Supporting Material 1 – Survey and Focus Group Questions. [file HEX-28-e70488-s004.docx]

Additional File 1 – Survey and Focus Group Questions

**Article:** “*Coproduction is Caring*”: Young People’s Reflections on Responsible and Dialogic Coproduction in Youth Mental Health

**Journal:** Health Expectations

**Authors:** Josimar Antônio de Alcântara Mendes; Sarah Doherty; Ayan Mahamud; Mathijs Lucassen; Joanna Lockwood; Chris Hollis; Ellen Townsend; Marina Jirotka

**Survey Open-ended Questions**

1. How would you define ‘mental health’?
2. In your opinion, what are the main determinants of adolescent mental health?
3. Regardless of your understanding or previous experiences with RRI, what do you think ‘Responsible Research and innovation’ (RRI) refers to? [Young People]
4. How do you think an RRI approach can be useful for projects focused on adolescent mental health?
5. What risks and/or unintended impacts/consequences do you think researchers should consider when they are addressing adolescent mental health? [Young People]
6. How do you think researchers could foresee and mitigate risks and/or unintended impacts/consequences when they are addressing adolescent mental health? [Young People]
7. In your opinion, what are the main advantages of collaborating with researchers in this project? [Young People]
8. In your opinion, what are the main challenges of collaborating with researchers in this project? [Young People]
9. What do you think is important whenever researchers and adults are collaborating with young people? [Young People]
10. How do you think a responsible approach could address diversity?
11. How do you think a responsible approach could address biases?

**Focus Groups Guiding Questions**

1. After watching the presentation, how do these results sound to you?
2. Do these results reflect your experiences as young people in the programme?
3. In light of the results on jargon and communication, how might these issues affect young people’s involvement?
4. Some researchers referred to the perceived “flakiness” of young people’s involvement. What are your views on this characterisation?
5. What are your reflections on the ‘kids’ and ‘grown-up’ tables culture?
6. The results suggest that RRI can support the involvement of young people in mental health research. What is your view on this?
