# Supplementary material for: ‘Co‐Production Is Caring’: Young People's Reflections on Responsible and Dialogic Co‐Production in Youth Mental Health
Source: Health Expect. 2025 Nov 25;28(6):e70488. doi: 10.1111/hex.70488 (PMC12646113; doi:10.1111/hex.70488)
Supplement: Supplementary file 2 — Supporting Material 2 – Reflexivity. [file HEX-28-e70488-s001.docx]

Supplementary Material 2 – Reflexivity

**Article:** “*Coproduction is Caring*”: Young People’s Reflections on Responsible and Dialogic Coproduction in Youth Mental Health

**Journal:** Health Expectations

**Authors:** Josimar Antônio de Alcântara Mendes; Sarah Doherty; Ayan Mahamud; Mathijs Lucassen; Joanna Lockwood; Chris Hollis; Ellen Townsend; Marina Jirotka

**Reflexivity by Dr Josimar Mendes**

My commitment to meaningful coproduction and youth participation is deeply rooted in both personal experience and professional trajectory. I grew up in a highly vulnerable and marginalised area on the outskirts of Brazil’s capital – commonly referred to as a ‘*favela*’^[[1]](#footnote-1)^. Despite the structural inequalities and adversities surrounding me, I was fortunate to engage in transformative social and educational opportunities that challenged prevailing expectations about what ‘*someone from my background*’ could achieve.

One pivotal moment occurred when I was just 7 years old. A professor from the University of Brasília was conducting a Community Psychology intervention in my neighbourhood. As part of the project’s home visit activities, the professor invited me to join the team – originally formed by undergraduate students – as a guide, since I knew the community as the palm of my hand. I used to sit in the front seat of the University’s Kombi, helping navigate the maze-like streets of the settlement. Over time, I was also included in planning sessions for community interventions. In hindsight, this was my first experience of “radical co-production”, one that planted early seeds of self-efficacy, validation, and social responsibility. This experience has remained foundational in shaping how I understand the importance of genuinely valuing young people’s perspectives and creating spaces where they are treated as equal contributors.

Since then, coproduction has remained central to my identity as both a person and a professional. After completing my PhD at the University of Sussex, I returned to Brazil to undertake a postdoctoral position that adopted a radical co-research methodology. This approach embedded young people and researchers in horizontal, co-constructed relationships throughout the project’s lifecycle. Over nearly two years, I witnessed first-hand the transformative power of this process – how meaningful, dialogic engagement could foster confidence, trust, and critical insight among young people, while also enhancing the quality and relevance of the interventions developed.

My subsequent work on Responsible Research and Innovation (RRI) in youth mental health research has reinforced my belief that children and young people are not only capable of contributing meaningfully to research, but are entitled to do so. Hence, I see them as subjects of rights, endowed with inherent creative and transformative capacities that can drive social justice and broader societal change. I have consistently advocated for frameworks that elevate these voices in responsible and inclusive ways.

Moreover, my academic background includes extensive research on the ‘principle of the best interests of the child’, with a focus on operationalising this principle for professionals working with children and adolescents, especially in legal and protection services settings. Across all these domains, my orientation towards reflexivity is shaped by a commitment to equity, critical engagement, and the pursuit of research that is not only rigorous, but also relational, responsive, and socially accountable.

1. ‘*Favela’* is the term commonly used in Brazil to refer to informal urban settlements characterised by precarious housing, limited access to public services, and historical social exclusion. These communities often emerge through self-construction and lack formal urban planning or legal land tenure, yet they are also sites of resilience, cultural production, and community organization. [↑](#footnote-ref-1)
