# Supplementary material for: ‘Co‐Production Is Caring’: Young People's Reflections on Responsible and Dialogic Co‐Production in Youth Mental Health
Source: Health Expect. 2025 Nov 25;28(6):e70488. doi: 10.1111/hex.70488 (PMC12646113; doi:10.1111/hex.70488)
Supplement: Supplementary file 3 — Supporting Material 3 – Initial Codes. [file HEX-28-e70488-s002.docx]

Supplementary Material 3 – Initial Codes

**Article:** “*Coproduction is Caring*”: Young People’s Reflections on Responsible and Dialogic Coproduction in Youth Mental Health

**Journal:** Health Expectations

**Authors:** Josimar Antônio de Alcântara Mendes; Sarah Doherty; Ayan Mahamud; Mathijs Lucassen; Joanna Lockwood; Chris Hollis; Ellen Townsend; Marina Jirotka

| Name | Description | Sources | References |
| --- | --- | --- | --- |
| **Coproduction is Caring** | This code captures young people’s understanding of coproduction as a caring, relational process. They described small but meaningful practices - like flexible communication, emotional check-ins, and sensitivity to lived experience - as essential to feeling respected and safe. Care was not seen as an optional extra, but as a core element that fostered trust and makes coproduction genuinely dialogic and collaborative | 6 | 16 |
| **Coproduction is a Dialogic Process** | This code captures young people’s understanding of coproduction as an ongoing dialogue rather than a one-way exchange. Participants valued opportunities for open conversation- such as away days, huddles, and informal chats - as spaces where mutual learning could occur. They highlighted the importance of researchers being present as people, not just professionals, and saw relational communication as essential to building trust, encouraging participation, and enhancing the quality of engagement | 5 | 12 |
| **Communication issues** | This code captures the importance of communication practices that enable genuine dialogue and mutual understanding. Participants highlighted efforts to reduce jargon, explain terminology, and create space for clarification as key features of respectful engagement. Rather than assuming shared knowledge, researchers were expected to ensure accessibility by adapting language and encouraging questions. Dialogic coproduction was seen as a process grounded in openness, clarity, and the ongoing negotiation of meaning | 4 | 11 |
| **Diversity** | This code captures participants’ reflections on the presence, challenges, and ongoing pursuit of diversity within the project. Young people recognised efforts to include varied experiences and backgrounds but also acknowledged areas of underrepresentation—such as gender, education, and employment status. Diversity was seen not as a static achievement but as a continual commitment, shaped by how intentionally and reflexively inclusion is approached during recruitment and participation | 4 | 10 |
| **Aiding the Coproduction Process** | This code captures practical and relational strategies that helped facilitate meaningful youth engagement in the coproduction process. Participants valued tools such as digital glossaries, handbooks, surveys, and follow-up emails, which supported understanding and allowed time for reflection. Structures like huddles and the Co-chair role were also seen as vital for relationship-building and fostering trust. These aids were understood as key enablers that made participation more inclusive, responsive, and effective | 5 | 8 |
| **Youth Challenging Researchers’ Perspectives** | This code captures how young participants reflected on their role in questioning and reshaping researchers’ assumptions. Rather than confrontational, these challenges were framed as constructive contributions that introduced nuance, offered alternative viewpoints, and bridged experiential and academic knowledge. Participants emphasised that respectful dialogue allowed for mutual learning, especially when researchers were open to seeing things differently. Challenging, in this context, was positioned as a collaborative act that enriched the research process | 5 | 8 |
| **‘Lived experience’ is a continuum** | This code captures participants’ critique of binary framings of lived experience. Young people questioned assumptions that lived experience is fixed in the past or held exclusively by certain groups. Instead, they described it as ongoing, shared, and context-dependent. They challenged rigid distinctions between 'researcher' and 'participant', 'young person' and 'professional', highlighting the fluidity of identity and experience. This perspective calls for more nuanced, inclusive understandings of expertise in coproduction | 2 | 6 |
| **Privacy issues** | This code captures participants’ reflections on privacy, consent, and data use within coproduction. Young people emphasised the importance of having clear choices over how their information is shared - such as anonymity, name usage, and data attribution. Comfort levels varied, highlighting that privacy is a personal and evolving concern. Trust, transparency, and ongoing consent were identified as essential to ensuring participants feel safe and respected when contributing to research | 4 | 6 |
| **Coproduction is a trade-off process** | This code captures how participants understood coproduction as a reciprocal exchange. Young people acknowledged both the personal benefits of involvement - such as learning opportunities, friendships, or a sense of purpose - and the value of their contributions to the research. Rather than viewing this exchange as problematic, they framed it as a healthy, transparent trade-off. Recognising and valuing what participants give and receive was seen as essential to sustaining meaningful engagement | 2 | 5 |
| **Being sensible to people with lived experiences** | This code captures reflections on the need for empathy, flexibility, and emotional awareness when working with people who bring lived experience to research. Participants noted that rigid structures and task-driven goals can overlook the human realities of involvement, particularly in mental health contexts. They stressed the importance of approaching sensitive topics with care, recognising the emotional demands of participation, and balancing project aims with compassion and understanding | 2 | 4 |
| **The Evolving Nature of Coproduction and the Need for Accommodations** | This code captures participants’ recognition that coproduction is an ongoing, adaptive process that requires flexibility and compromise. They acknowledged the tension between research structures - such as deadlines and deliverables - and the realities of involving people with lived experience. Rather than expecting perfection, participants emphasised the importance of openness, understanding, and the willingness to adjust expectations to support meaningful collaboration over time | 2 | 4 |
| **Us vs Them** | This code captures young people’s reflections on the perceived divide between researchers and youth participants. While some structural distinctions - such as roles or labels - can help formalise inclusion, they can also reinforce a sense of separation. Participants questioned how useful or necessary these divisions are, especially when lived experiences overlap across roles. They called for greater efforts to bridge these divides and foster mutual understanding, without erasing differences or reinforcing hierarchies | 2 | 4 |
| **Room for disagreement** | This code captures the value participants placed on having space to express disagreement within the coproduction process. Young people appreciated environments where they could challenge ideas openly and without repercussion, viewing this as a rare and empowering experience. The ability to disagree respectfully was seen as essential to genuine collaboration, reinforcing the idea that all contributions were welcomed and that consensus was not expected or imposed | 1 | 2 |
| **The role of young people** | This code captures the significance of young people's roles within the project, particularly through positions like the Co-chair. Participants viewed these roles as crucial in bridging the gap between researchers and youth, facilitating mutual understanding and participation. Young people in these positions were not only seen as representatives but as active enablers of engagement, whose presence made others feel more comfortable and willing to take part | 1 | 2 |
| **Coproduction and self-accomplishment** | This code captures the sense of personal fulfilment and growth that young people associated with their involvement in coproduction. Participation was not only about contributing to research but also about gaining something meaningful for oneself—such as confidence, comfort, or a sense of achievement. Coproduction was experienced as a space where young people could feel valued and supported in ways that contributed to their own development | 1 | 1 |
| **Due recognition** | This code captures the importance of acknowledging young people’s contributions through meaningful forms of recognition, such as co-authorship. Participants valued being credited not just as contributors, but as collaborators, with opportunities to be named in outputs like research papers. This recognition reinforced their sense of value within the project and reflected a commitment to equitable and respectful coproduction practices | 1 | 1 |
| **Learning from mistakes** | This code captures participants’ interest in reflecting on moments where coproduction did not work as intended. Rather than focusing solely on success stories, young people expressed a desire to understand challenges and setbacks - what went wrong, why, and how to improve. They saw value in openly discussing difficulties as a way to foster learning, growth, and more honest, responsive coproduction practices | 1 | 1 |
| **Young people are more than their 'lived experiences'** | This code captures the idea that young people involved in coproduction should not be reduced to their lived experiences alone. Participants emphasised the importance of being recognised as multifaceted individuals, with identities, interests, and lives that extend beyond the roles assigned to them in research. This perspective challenges narrow or tokenistic framings and supports more holistic, person-centred engagement | 1 | 1 |
