# Supplementary material for: ‘Co‐Production Is Caring’: Young People's Reflections on Responsible and Dialogic Co‐Production in Youth Mental Health
Source: Health Expect. 2025 Nov 25;28(6):e70488. doi: 10.1111/hex.70488 (PMC12646113; doi:10.1111/hex.70488)
Supplement: Supplementary file 4 — Supporting Material 4 – Candidate Themes. [file HEX-28-e70488-s003.docx]

Supplementary Material 4 – Candidate Themes

**Article:** “*Coproduction is Caring*”: Young People’s Reflections on Responsible and Dialogic Coproduction in Youth Mental Health

**Journal:** Health Expectations

**Authors:** Josimar Antônio de Alcântara Mendes; Sarah Doherty; Ayan Mahamud; Mathijs Lucassen; Joanna Lockwood; Chris Hollis; Ellen Townsend; Marina Jirotka

| **Number of candidate themes**: 2  **Number of features**: 6 |
| --- |

# Candidate Theme 1: The Care Dimension of Coproduction

| **General description**: this theme captures young people’s understanding of coproduction as a process rooted in emotional sensitivity, ongoing care, and mutual respect. It highlights how caring is enacted through empathy, responsiveness, and inclusivity. Young people emphasised that coproduction should acknowledge their ongoing lived experiences, human vulnerabilities, and the need to feel safe, heard, and respected. Rather than treating participation as purely instrumental, care is experienced as a relational and ethical commitment within the research and collaboration process |
| --- |

Feature (a): Recognising ‘lived experience’ as a common and ongoing issue

***Description***: this feature reflects young people’s recognition that ‘lived experience’ is not a fixed or past event, but something dynamic and ongoing. Coproduction should consider that young people continue to navigate the same challenges they are being invited to discuss

***Source:*** [YP1-FC, YP2-FC]

Feature (b): Being sensible about ‘lived experience’

***Description***: this feature highlights how young people want their participation to be approached with sensitivity and care, particularly given the personal and emotional nature of the topics discussed. They want researchers to remember that they are not just ‘sources of data’, but people with ongoing lives, vulnerabilities, and mental health needs

***Source:*** [YP1-FC, YP2-FC, YP4-FC, YP5-FC]

Feature (c): Co-production as a responsive, safe and inclusive space

***Description***: this feature captures young people’s emphasis on the need for coproduction spaces to be flexible, inclusive, and welcoming. They valued environments where questions are encouraged, wellbeing is prioritised, and participation could occur in multiple formats. A sense of safety, respect, and mutual care was central to feeling genuinely involved

***Source:*** [YP1-FC, YP2-FC, YP3-FC, YP4-FC, YP5-FC, YP10-S, YP13-S]

# Candidate Theme 2: Coproduction is Dialogic: Coproduction as Dialogic Process

| **General description**: this theme captures how young people see coproduction as a dialogic and reciprocal process that involves ongoing conversation, mutual learning, and recognition of personal and collective contributions. Rather than one-way communication or rigid role separation, coproduction thrives when space is created for genuine exchange, informality, and shared ownership of meaning and knowledge |
| --- |

Feature (a): Us vs. Them

***Description***: this feature addresses how young people perceive the division between ‘researchers’ and ‘young people’ roles, especially when fixed roles are emphasised. While distinctions (e.g., ‘researcher’ vs. ‘young person’) can create barriers, young people also recognise the practical need for role definition. Importantly, they call for reflexivity about these boundaries and openness to more fluid and relational roles

***Source:*** [YP1-FC, YP2-FC, YP5-FC, YP7-FC]

Feature (b): Aiding the dialogic process

***Description***: this feature reflects young people’s appreciation of mechanisms that enable ongoing, dialogic interaction – such as huddles, co-chairs, away days, and feedback loops. These structures help sustain engagement, build relationships, and accommodate different forms of participation

***Source:*** [YP1-FC, YP2-FC, YP3-FC, YP4-FC, YP5-FC, YP6-FC]

Feature (c): Room for exchange and learning

***Description***: this feature centres on the importance of informal, humanising dialogue in coproduction. Young people valued when researchers showed their personalities and engaged beyond their roles. Small talk, jokes, and vulnerability made the environment more comfortable and encouraged open, reflective conversations

***Source:*** [YP1-FC, YP2-FC, YP3-FC, YP4-FC, YP5-FC, YP9-S, YP12-S]
